# MicroRNA-221 and microRNA-222 regulate gastric carcinoma cell proliferation and radioresistance by targeting PTEN

**DOI:** 10.1186/1471-2407-10-367

**Published:** 2010-07-12

**Authors:** Zhang Chun-zhi, Han Lei, Zhang An-ling, Fu Yan-chao, Yue Xiao, Wang Guang-xiu, Jia Zhi-fan, Pu Pei-yu, Zhang Qing-yu, Kang Chun-sheng

**Affiliations:** 1 Department of Neurosurgery, Tianjin Medical University General Hospital and Lab of Neuro-oncology, Tianjin Neurological Institute, Tianjin 300052, China; 2 Department of Radiation Oncology, Tianjin Huan Hu Hospital, Tianjin 300060, China; 3 Department of Gastroenterology, Tianjin Medical University General Hospital, Tianjin 300052, China

## Abstract

**Background:**

MicroRNAs (miRNAs) can function as either oncogenes or tumor suppressor genes via regulation of cell proliferation and/or apoptosis. MiR-221 and miR-222 were discovered to induce cell growth and cell cycle progression via direct targeting of p27 and p57 in various human malignancies. However, the roles of miR-221 and miR-222 have not been reported in human gastric cancer. In this study, we examined the impact of miR-221 and miR-222 on human gastric cancer cells, and identified target genes for miR-221 and miR-222 that might mediate their biology.

**Methods:**

The human gastric cancer cell line SGC7901 was transfected with AS-miR-221/222 or transduced with pMSCV-miR-221/222 to knockdown or restore expression of miR-221 and miR-222, respectively. The effects of miR-221 and miR-222 were then assessed by cell viability, cell cycle analysis, apoptosis, transwell, and clonogenic assay. Potential target genes were identified by Western blot and luciferase reporter assay.

**Results:**

Upregulation of miR-221 and miR-222 induced the malignant phenotype of SGC7901 cells, whereas knockdown of miR-221 and miR-222 reversed this phenotype via induction of PTEN expression. In addition, knockdonwn of miR-221 and miR-222 inhibited cell growth and invasion and increased the radiosensitivity of SGC7901 cells. Notably, the seed sequence of miR-221 and miR-222 matched the 3'UTR of PTEN, and introducing a PTEN cDNA without the 3'UTR into SGC7901 cells abrogated the miR-221 and miR-222-induced malignant phenotype. PTEN-3'UTR luciferase reporter assay confirmed PTEN as a direct target of miR-221 and miR-222.

**Conclusion:**

These results demonstrate that miR-221 and miR-222 regulate radiosensitivity, and cell growth and invasion of SGC7901 cells, possibly via direct modulation of PTEN expression. Our study suggests that inhibition of miR-221 and miR-222 might form a novel therapeutic strategy for human gastric cancer.

## Background

Gastric cancer, a highly invasive and aggressive malignancy that is characterized by resistance to apoptosis and radioresistance, is among the most common cancers and is the leading cause of cancer-related death in China [1-6]. Gastric cancer in China is often diagnosed at an advanced clinical stage, with evident lymphatic tumor dissemination [4]. The 5-year survival rate is approximately 60% for patients with localized disease, but only 2% for those with metastatic disease[7]. Although much has been learned about the genetic and biochemical bases of gastric cancer, few novel therapeutic targets have been identified, due to difficulties in target identification and validation.

MicroRNAs (miRNAs) are noncoding RNAs of approximate 22 nt in length that function as post-transcriptional regulators. By base-pairing with the complementary sites in the 3'untranslated region (3'UTR) of the mRNA, miRNAs control mRNA stability and translation efficiency [8-12]. Growing evidence indicates the important role of miRNA in the development of various cancers. Deregulation of some miRNAs, including miR-221 and miR-222, have been observed in lymphoma, colorectal, lung, and breast cancers, papillary thyroid and hepatocellular carcinoma, glioblastoma [13-21], and gastric cancer [22,23].

The PTEN gene, located at 10q23.3, encodes a central domain with homology to the catalytic region of protein tyrosine phosphatases. This gene is an important regulator of protein phosphatases and 3'-phosphoinositol phosphatases. PTEN dephosphorylates phosphatidylinositol-3,4,5-triphosphate (PIP3), the second messenger produced by phosphoinositide 3-kinase (PI3K), to negatively regulate the activity of the serine/threonine protein kinase, Akt [24,25]. PTEN is inactivated in some malignant tumors, resulting in Akt hyper-activation, thereby promoting cell proliferation, inhibition of apoptosis, and enhanced cell invasion and radioresistance [26-28]. miRNA, specifically miR-21 and miR-214, have been established as regulators of PTEN expression [29-33].

In the current study, we predicted that PTEN would be a target gene of the miR-221 and miR-222 cluster by computer-aided algorithm. Moreover, we found binding sites for human miR-221 and miR-222 in the PTEN 3'-UTR. Based upon these findings, we confirmed PTEN as a target of miR-221 and miR-222, and demonstrated that co-suppression of the miR-221/222 cluster inhibits cell proliferation, induces cell apoptosis, inhibits cell invasion and enhances cell radiosensitivity by upregulating PTEN expression in SGC7901 gastric cancer cells.

## Methods

### Cells and cell culture

The human gastric cancer cell line SGC7901 was kindly provided by Dr. Daiming Fan (the Fourth Military Medical University, China). The human embryonic kidney cell line HEK293 was obtained from the Institute of Biochemistry and Cell Biology, Chinese Academy of Sciences. Cells were grown in Dulbecco's Modified Eagle's medium (DMEM; Gibco, USA) supplemented with 10% fetal bovine serum at 37°C in 5% CO_2 _atmosphere.

### Identification of microRNA targets

The PicTar algorithm http://pictar.mdc-berlin.de. was used to identify human microRNA binding sites in PTEN (GeneID 5728). Briefly, PicTar provides 3' UTR alignments with predicted sites and links to various public databases for prediction of microRNA binding sites.

### Plasmids, oligonucleotides and cell transfection

Human full-length miR-221 and miR-222 in pMSCV vector were kindly provided by Reuven Agami (Division of Tumor Biology, The Netherlands Cancer Institute, Amsterdam, Netherlands). The recombinant retroviruses pMSCV-miR-221 and pMSCV-miR-222 were produced as previously described [34], and transfected into PT67, the packaging cells, using Lipofectamine 2000. The titers of homogenous virus were calculated after infection of NIH3T3 cells. Wild-type PTEN lacking the 3'UTR region was constructed in the pcDNA vector (pcDNA-PTEN) by Genesil Biotechnology Co. Ltd. (Wuhan, China). 2'-OMe-oligonucleotides were chemically synthesized by GenePharma Co. Ltd. (Shanghai, China). All the bases were 2'-OMe modified and had the following sequences: 2'-OMe-anti-miR-221 (AS-miR-221), 5'-AGCUACAUUGUCUGCUGGGUUUC-3'; 2'-OMe-anti-miR-222 (AS-miR-222), 5'-AGCUACAUCUGGCUACUGGGU-3'; scrambled oligonucleotide (Scr), 5'-UCUA CUCUUUCUAGGAGGUUGUGA-3'.

SGC7901 cells were grown to 70-80% confluence and transfected with pcDNA- PTEN and 2'-OMe-oligonucleotides using Lipofectamine 2000 or infected with pMSCV-miR-221 and/or pMSCV-miR-222 at a multiplicity of infection (MOI) of 50 at 37°C. At 4 h after infection, the medium was replaced with fresh DMEM containing 10% fetal bovine serum, and the cells were incubated for an additional 72 h for further study.

### Northern blot analysis

Total RNA was extracted using TRIzol reagent (Invitrogen). The protocol for Northern blotting of miRNA was adopted from Ramkissoon [35]. Total RNA were separated on a 12% denaturing polyacrylamide gel and transferred to Hybond N+ nylon membrane (Ambion, USA). The membrane was dried, UV cross-linked, hybridized with digoxigenin (DIG)-labeled probes overnight at 37°C in a buffer containing 5× SSC, 20 mmol/L Na_2_HPO_4 _(pH = 7.2), 7% SDS, 1× Denhardt's solution and 0.2 mg/mL salmon sperm DNA. The specific probes, end-labeled with DIG, were miRNA-221, 5'-GAAACCCAGCAGACAATGTAGCT-3'; miRNA-222, 5'-GAGACC CAGTAGCCAGATGTAGCT-3'; and U6, 5'-ATTTGCGTGTCATCCTTGCG-3'. The probes were purchased from Proligo Primers & Probes (Sigma, USA). Membranes were washed with 1× SSC/1% SDS at 50°C. After equilibration in detection buffer, blots were detected with a DIG Luminescent Detection Kit (Roche, USA) and analyzed using GeneGenius.

### Cell viability assay

Cells were seeded into 96 well plates at 4000 cells/well. After transfection, 20 μl MTT (5 mg/mL) was added into a corresponding test well, and incubated for 4 h. The supernatant was then discarded, and 200 μL of DMSO was added to each well to dissolve the precipitate. Optical density (OD) was measured at the wavelength of 570 nm. Each test was performed daily for six consecutive days and repeated in eight wells.

### Cell cycle assay

For cell cycle analysis, parental and transfected cells in the log phase of growth were stained with propidium iodide and examined with a fluorescence-activated cell-sorting (FACS) flow cytometer (BD Biosciences, San Jose, CA), and DNA histograms were analyzed with modified software. Each test was repeated in triplicate.

### Measurement of early apoptosis by Annexin V staining

Parental and transfected cells in the log phase of growth were harvested and collected by centrifugation and resuspended at a density of 1 × 10^6 ^cells/mL. For the apoptosis assay, an annexin V-FITC labeled Apoptosis Detection Kit (Abcam, USA) was used. The pre-labeled cells were detected and apoptosis was quantified using a FACSCalibur flow cytometer (Becton-Dickinson, USA). The data obtained were analyzed using CellQuest software. Each test was repeated in triplicate.

### Invasion Assay

Using parental and transfected cells, the invasion potential of the cells were evaluated by measuring the number of cells invading Matrigel-coated Transwell chambers (Becton Dickinson). Transwell inserts with 8 μm pores were coated with Matrigel and reconstituted with fresh medium for 2 h before the experiment. Cells (2 × 10^4^/mL) were seeded into the upper chambers in 250 μL serum free DMEM, while DMEM supplemented with 10% fetal bovine serum (750 μL) was placed in the lower chamber. Cells were incubated for 72 h. Cells that degraded the Matrigel and invaded the lower surface of the Matrigel-coated membrane were fixed with 70% ethanol, stained with hematoxylin and counted in five random fields at ×200 magnification under a light microscope. The results were expressed as the average number of invasive cells per field.

### Radiation Exposure and Clonogenic assay

Irradiation was performed at room temperature in a linear accelerator (Varian600, Varian, USA) at a dose rate of 3.2 Gy/min. Cells were plated into six-well plates and exposed to the specified dose (0, 2, 4 and 6 Gy) of X-rays. At 24 h after irradiation, all cells were trypsinized and counted. Corresponding numbers of cells were seeded into 10 cm dishes containing DMEM supplemented with 10% fetal bovine serum in triplicate, incubated for 10-14 days to allow colony growth, and colonies were stained with crystal violet. Colonies containing 50 or more cells were counted. The plating efficiency was calculated by dividing the average number of colonies per dish by the number of cells plated. Survival fractions were calculated by normalization to the plating efficiency of appropriate control groups.

### Luciferase reporter assay

The human 3'-UTR of the PTEN gene was amplified by PCR using the following primers: PTEN-3'UTR-Forward: 5'-CGATTCTAGAAATCATGTTCTGGTGG-3' and PTEN-3'UTR-Reverse: 5'-GCATTCTAGAATTCTGCACAGTAAGCATA-3'. The cDNA was cloned into the XbaI/XbaI site of the pGL3-control vector (Promega, USA), downstream of the luciferase gene, to generate the vector pGL3-PTEN. For the luciferase reporter assay, SGC7901 cells were cultured in 96-well plates, transfected with 0.2 μg of the pGL3-PTEN or pGL3-control plasmids and 5 pmol of AS-miRNAs (AS-miR-221 and/or AS-miR-222) using Lipofectamine 2000. At 48 h after transfection, luciferase activity was measured using the Luciferase Assay System (Promega).

### Western blot analysis

Parental and transfected cells were washed with pre-chilled PBS and solubilized in 1% Nonidet P-40 lysis buffer. Homogenates were clarified by centrifugation at 20,000 ×g for 15 min at 4°C and the protein concentration was measured by bicinchoninic acid protein assay kit (Pierce Biotechnology). 40 μg of protein from each sample was subjected to SDS-PAGE on SDS-acrylamide gel. Separated proteins were transferred to PVDF membranes (Millipore) and incubated with primary antibody (1:1000 dilution; Santa Cruz) followed by incubation with an HRP-conjugated secondary antibody (1:1000 dilution; Zymed, San Diego, CA). The specific protein was detected using a SuperSignal protein detection kit (Pierce, USA). The membrane was stripped and reprobed with a primary antibody against β-actin (Santa Cruz; 1:1000 dilution) as a control.

### Statistical Analysis

Data are expressed as the mean ± standard error (S.E.). P < 0.01 was considered statistically significant using ANOVA and the STD t test or SNK Q test t test.

## Results

### Modulation of miR-221 and miR-222 expression in SGC7901 cell lines

**Figure 1:**
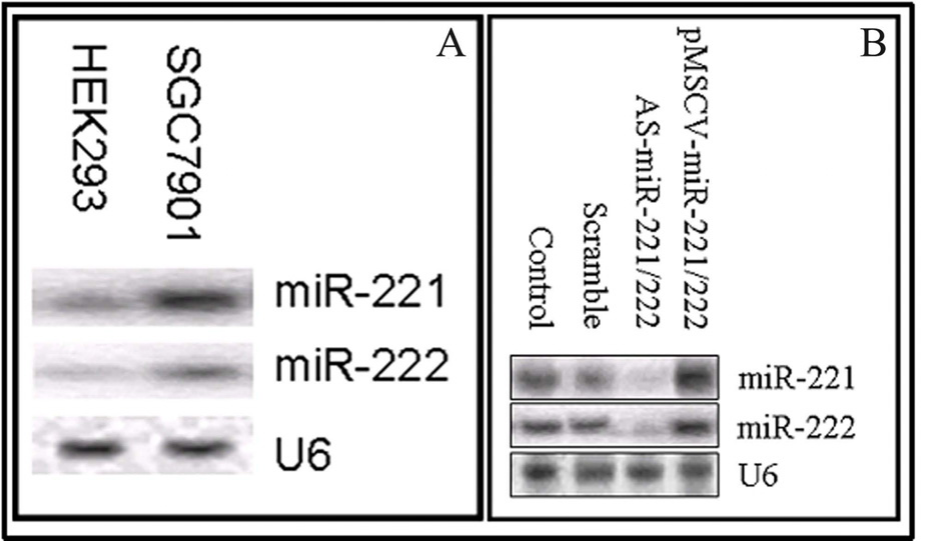
**miR-221 and miR-222 expression in SGC7901 and HEK293 cells**. Northern blot showing that expression of miR-221 and miR-222 in SGC7901 cells was greater than that in HEK293 cells (Figure 1A). pMSCV-miR-221/222 infection increased the expression of miR-221 and miR-222, while AS-miR-221/222 transfection efficiently silenced the expression of miR-221 and miR-222 in SGC7901 cells (Figure 1B).

Sequence analysis predicted that miR-221 and miR-222 would regulate PTEN expression. To determine the biologic impact of miR-221 and miR-222 in the SGC7901 gastric cancer line, cells were transfected with AS-miR-221/222 or infected with pMSCV-miR-221/222 to reduce or increase miRNA levels, respectively. Northern blot analysis revealed that the expression of miR-221 and miR-222 was greater in SGC7901 cells than in normal kidney epithelial HEK293 cells (Figure 1A). HEK293 cells were used as negative control in these studies since both HEK293 and SGC7901 cells belong to the epithelial cells [36]. Infection of SGC7901 cells with pMSCV-miR-221/222 increased miR-221 and miR-222 expression, while transfection with AS-miR-221/222 efficiently silenced miR-221 and miR-222 expression in this cells (Figure 1B). These strategies were then used as the basis of the remaining experiments.

### miR-221 and miR-222 co-modulate SGC7901 cell proliferation

**Figure 2:**
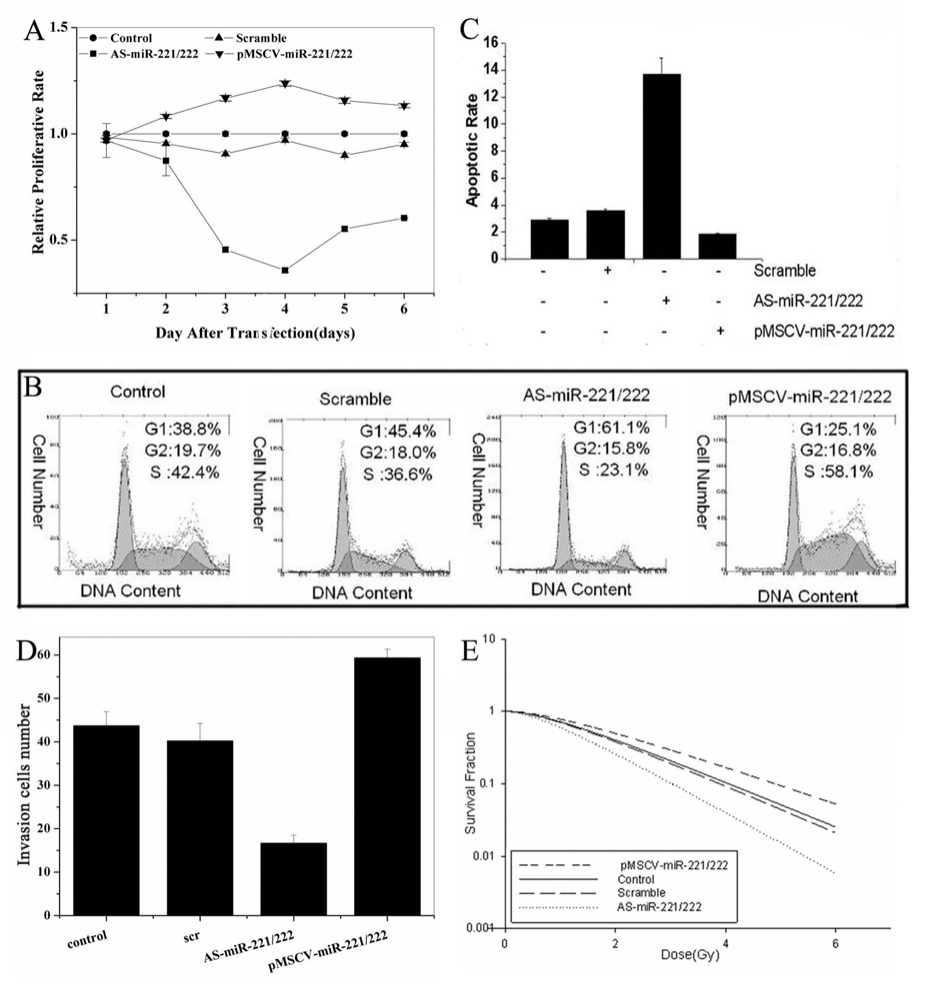
**miR-221 and miR-222 affect phenotype of SGC7901 cells**. MTT assay showing that cells transfected with AS-miR-221/222 proliferated at a significantly lower rate than controls (p = 0.0023). However, the cells infected with pMSCV-miR-221/222 proliferated at a significantly higher rate than controls (p = 0.0311) (Figure 2A). Flow cytometry analysis showing that the proportion of cells in the G1 phase in the AS-miR-221/222 group was significantly higher than that in the control group (p = 0.0036) and that the proportion of cells in the G1 phase in the pMSCV-miR-221/222 group was significantly lower than in the control group (p = 0.0266) (Figure 2B). Annexin V analysis showing that the cells transfected with AS-miR-221/222 underwent apoptosis at a significantly higher rate than controls(p = 0.0012), while cells infected with pMSCV-miR-221/222 underwent apoptosis at a significantly lower rate than controls (p = 0.0198) (Figure 2C). Transwell assay showing a decrease in invasive ability in the AS-miR-221/222 group and an increase in the pMSCV-miR-221/222 group compared to controls (Figure 2D). Clonogenic assay indicating that the radiosensitivity of SGC7901 cells increased in the AS-miR-221/222 group (p = 0.0032) and decreased in the pMSCV-miR-221/222 group compared with controls (p = 0.043) (Figure 2E).

The proliferation rates of SGC7901 cells with enhanced or silenced expression of miR-221 and miR-222 was determined via MTT assay. Compared to control and scramble-transfected cells, cells transfected with AS-miR-221/222 proliferated at a significantly lower rate. In contrast, overexpression of miR-221 and miR-222 by infection with pMSCV-miR-221/222 resulted in significantly enhanced proliferation (Figure 2A). Cell cycle distribution by flow cytometry yielded similar results (Figure 2B). The percentage of control and scramble treated cells in the G0/G1 phase was 38.8 ± 2.2% and 45.4 ± 1.2%, respectively, while AS-miR-221/222 transfection and pMSCV-miR-221/222 infection resulted in 61.1 ± 3.4% and 25.1 ± 0.9% of cells in G0/G1, respectively. The S phase fraction in control, scramble, AS-miR-221/222 and pMSCV-miR-221/222 groups were 42.2 ± 2.3%, 36.6 ± 1.7%, 23.1 ± 0.8% and 58.1 ± 3.1%, respectively. In sum, transfection with AS-miR-221/222 resulted in the highest percentage of cells in G0/G1 phase (p = 0.0036), and lowest fraction in S phase (p = 0.0031). No statistical significance was observed in the percentage of cells in the G2/M phase among the four groups.

Apoptosis is a genetically encoded cascade of cellular reaction that results in the disposal of unwanted cells. Disruption to this pathway has been implicated as a cause of cancer [37]. Some miRNAs regulate proteins that are involved in apoptosis [38]. Using Annexin V analysis, the number of apoptotic cells in early phase was found to be significantly increased in cells transfected with AS-miR-221/222 compared with that in other groups (p = 0.0012). In comparison with parental cells, the apoptotic rate was very low in pMSCV-miR-221/222 infected cells (Figure 2C). These data demonstrated that the proliferation and survival rates of SGC7901 cells might be co-modulated by miR-221 and miR-222.

### miR-221 and miR-222 co-modulate SGC7901 cell invasion

We also assessed the role of miR-221 and miR-222 on cell invasion by Transwell assay. As shown in Figure 2D, as compared with blank and negative control cells, the invasion potential of SGC7901 cells transfected with AS-miR-221/222 was significantly decreased (0.3813-fold, p = 0.0067), while cells transduced with pMSCV-miR-221/222 displayed markedly increased invasive ability (1.3577-fold, P = 0.0099). These results suggested that miR-221 and miR-222 could co-modulate SGC7901 cell invasion.

### miR-221 and miR-222 co-modulate SGC7901 cell radiosensitivity

The national comprehensive cancer network guidelines on gastric cancer treatment include radiotherapy as a standard treatment for patients with a high risk of recurrence http://www.nccn.org/index.asp. To determine whether miR-221 and miR-222 affected SGC7901 cell radiosensitivity, cells were transfected with AS-miR-221/222 or infected with pMSCV-miR-221/222 and colony formation was assessed following 0-6 Gy radiation (Figure 2E), Transfection of SGC7901 cells with AS-miRNA-221/222 significantly decreased survival following radiation exposure. Conversely, infection of SGC7901 cells with pMSCV-miR-221/222 significantly increased survival following 0-6 Gy compared to blank and negative control. The D_0 _value, the radiation dose required to reduce the level of cell survival from 100% to 37%, which is considered a measure of the intrinsic radiosensitivity of the cell, was calculated following genetic manipulation of miR-221/222. Control cells, cells transfected with scrambled oligonucleotides or AS-miRNA-221/222 or cells infected with pMSCV-miR-221/222 exhibited D_0 _values of 1.3897 Gy, 1.3326 Gy, 1.0358 Gy and 1.6770 Gy, respectively. The sensitization enhancement ratio (SER), calculated by determining the ratio of the D_0 _of the control group *vs*. treated cells, was 1.0428, 1.3417 and 0.8287 for scramble-, AS-miRNA-221/222-, or pMSCV-miR-221/222-treated cells, respectively (Table 1). Collectively, these results provide strong evidence that miRNA-221/222 co-regulates the radiosensitivity of SGC7901 cells.

**Table 1:** Impact of miRNA221/222 expression on SGC7901 cell radiosensitivity.

Group	D_0_	D_q_	SF_2_	SER
**control **+ **irradiation**	1.3897	2.6293	0.3865	
**Scrambled **+ **irradiation**	1.3326	2.5213	0.3639	1.0428
**AS-miRNA221/222 **+ **irradiation**	1.0358	1.9597	0.25	1.3417
**pMSCV-miR-221/222 **+ **irradiation**	1.677	3.1729	0.4536	0.8287

SGC7901 cells were transfected with scrambled or AS-miRNA221/222 or infected with pMSCV-miR-221/222. D_0 _and D_q _were determined by standardized software, and the sensitization enhancement ratio (SER) was calculated by determining the ratio of the D_0 _of the control group *vs*. treated cells.

### miR-221 and miR-222 targeting of the PTEN gene

**Figure 3:**
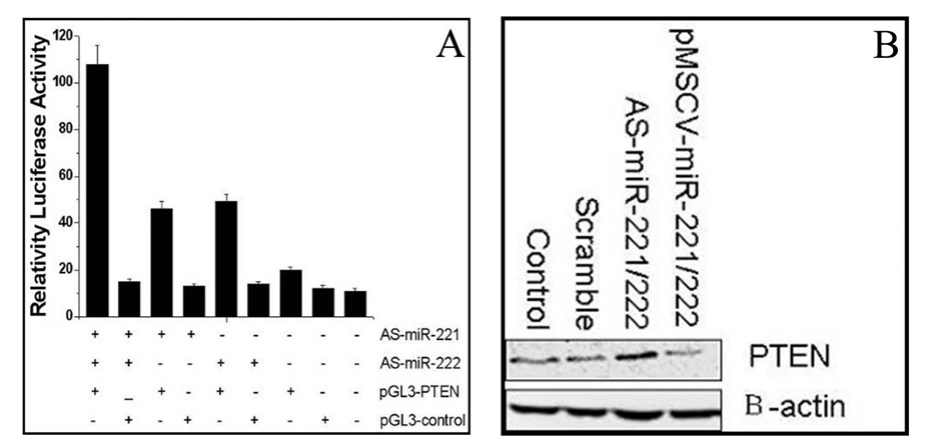
**PTEN is a target gene of miR-221 and miR-222**. pGL3-PTEN construct containing PTEN 3'UTR was transfected into SGC7901 cells previously transfected with AS-miR-221 and/or AS-miR-222. Luciferase activity was determined 48 h after transfection. The ratio of normalized sensor to control luciferase activity is shown. Error bars represent the standard deviation and were obtained from three independent experiments (Figure 3A). Western blot analysis demonstrating that PTEN expression was significantly enhanced in AS-miR-221/222 treated cells, and significantly downregulated in the pMSCV-miR-221/222 infected group compared to controls (Figure 3B).

Using bioinformatics analysis, we found that miR-221 and miR-222 contained specific binding sequences for the 3'UTR region of the PTEN gene. To confirm that PTEN is a target of miR-221 and miR-222, we cloned the PTEN 3'UTR fragment containing the putative miR-221/222 target site into pGL3-control vector with a luciferase reporter gene (pGL3-PTEN). As shown in Figure 3A, co-transfection of AS-miR-221/222 with pGL3-PTEN significantly enhanced luciferase activity compared to scramble or control treated cells (p = 0.0011). Furthermore, Western blot analysis showed that PTEN was significantly upregulated in AS-miR-221/222 transfected cells. In contrast, PTEN expression was downregulated in pMSCV-miR-221/222 infected cells (Figure 3B). Together, these data demonstrated that PTEN is a target gene of the miR-221/222 cluster.

### miR-221 and miR-222 affect the phenotype of SGC7901 cell in a PTEN-dependent pattern

**Figure 4:**
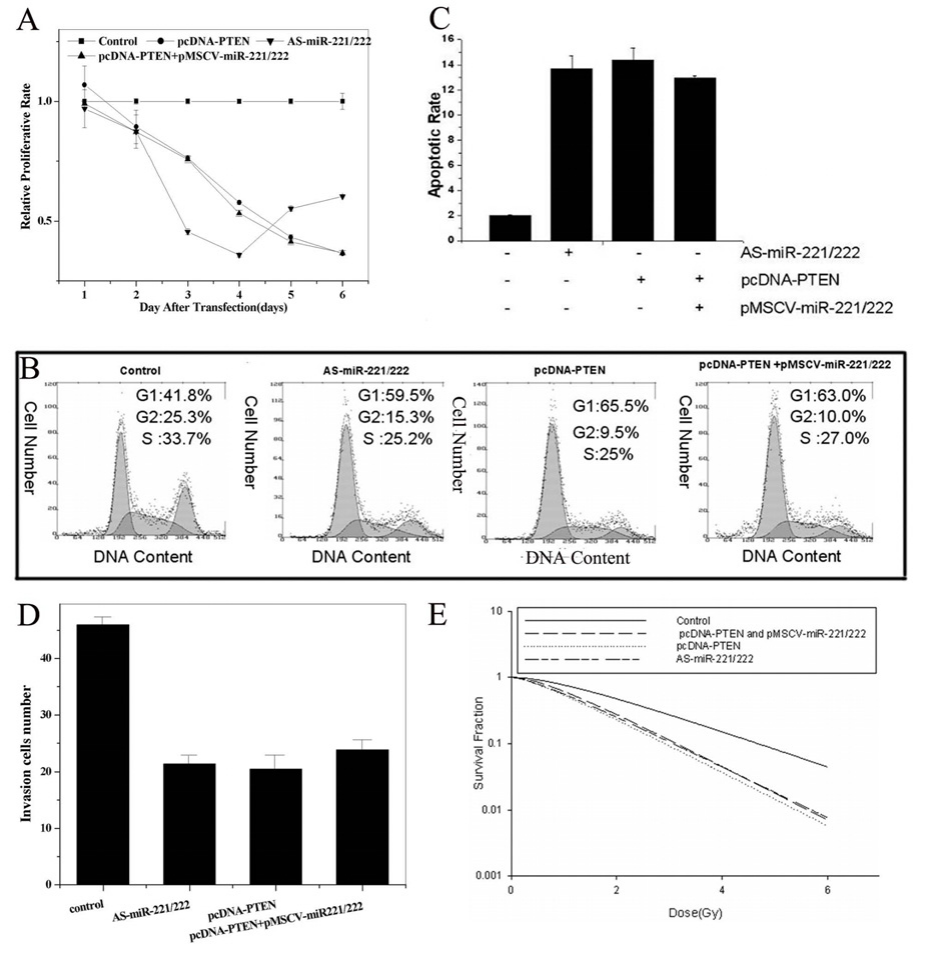
**PTEN regulates the impact of miR-221/222**. SGC7901 cells were cultured and treated with pcDNA-PTEN, AS-miR-221/222, or pMSCV-miR-221/222, and subjected to MTT assay. Transfection with pcDNA-PTEN increased cell proliferation to a similar rate as cells transfected with AS-miR-221/222. Infection of pcDNA-PTEN-treated cells with pMSCV-miR-221/222 had no effect on proliferation (Figure 4A). Flow cytometry analysis showing that the fraction of cells in G1 phase following AS-miR-221/222 transfection was significantly higher than in the control. Transfection with pcDNA-PTEN resulted in statistically similar results as with AS-miR-221/222, and infection of pcDNA-PTEN transduced cells with pMSCV-miR-221/222 did not impact on cell cycle progression (Figure 4B). Annexin V staining and flow cytometry analysis showing that AS-miR-221/222 transfection induced significantly higher levels of apoptosis in SGC7901 cells than controls, and transfection with pcDNA-PTEN yielded similar results. Infection of pcDNA-PTEN-transfected cells with pMSCV-miR-221/222 did not impact on apoptosis (Figure 4C). Transwell assay showing that AS-miR-221/222 transfection decreased invasive ability compared to controls. Transfection with pcDNA-PTEN yielded similar results, and infection of pcDNA-PTEN-transfected cells with pMSCV-miR-221/222 did not impact on invasive ability. Data represents the number of migrated cells per field (Figure 4D). AS-miR-221/222 and pcDNA-PTEN transfection increased radiosensitivity, as determined by clonogenic assay following radiation exposure. Infection of pcDNA-PTEN-transfected cells with pMSCV-miR-221/222 did not impact clonogenic survival. Experiments were performed in triplicate. When applicable, data is represented as mean ± SE (Figure 4E).

To determine the role of PTEN in miR-221 and miR-222 co-regulation of the SGC7901 phenotype, cells were transfected with pcDNA-PTEN. We observed a similar phenotype in pcDNA-PTEN transfected cells as observed in cells transfected with AS-miR-221/222, including decreased viability, enhanced apoptosis, prolonged G0/G1 phase transition, and reduced cell invasive capacity (Figure 4A-E). As the pcDNA-PTEN construct does not include the 3'UTR region of PTEN, transduction SGC7901 cells with both pcDNA-PTEN and pMSCV-miR-221/222 had no impact on viability, apoptosis, cell cycle progression, and invasive ability compared to transfection with pcDNA-PTEN alone (Figure 4A-E and Table 2). These results define an important role for PTEN as a mediator of the biological effects of miR-221/222 in SGC7901 gastric cancer cells.

**Table 2:** Impact of PTEN on miRNA221/222-mediated SGC7901 cell radiosensitivity.

Group	D_0_	D_q_	SF_2_	SER
**control **+**irradiation**	1.6031	3.0331	0.4566	
**pcDNA-PTEN +irradiation**	1.0719	1.6422	0.2230	1.4956
**AS-miR-221/222 **+**irradiation**	1.0723	1.6428	0.2436	1.4950
**pMSCV-miR-221/222 and pcDNA-PTEN + irradiation**	1.1303	1.7316	0.2630	1.4183

SGC7901 cells were transfected with pcDNA-PTEN or AS-miRNA221/222 or infected with pMSCV-miR-221/222 and transfected with pcDNA-PTEN. D0, Dq and SER were determined as described in Table 1.

**Figure 5:**
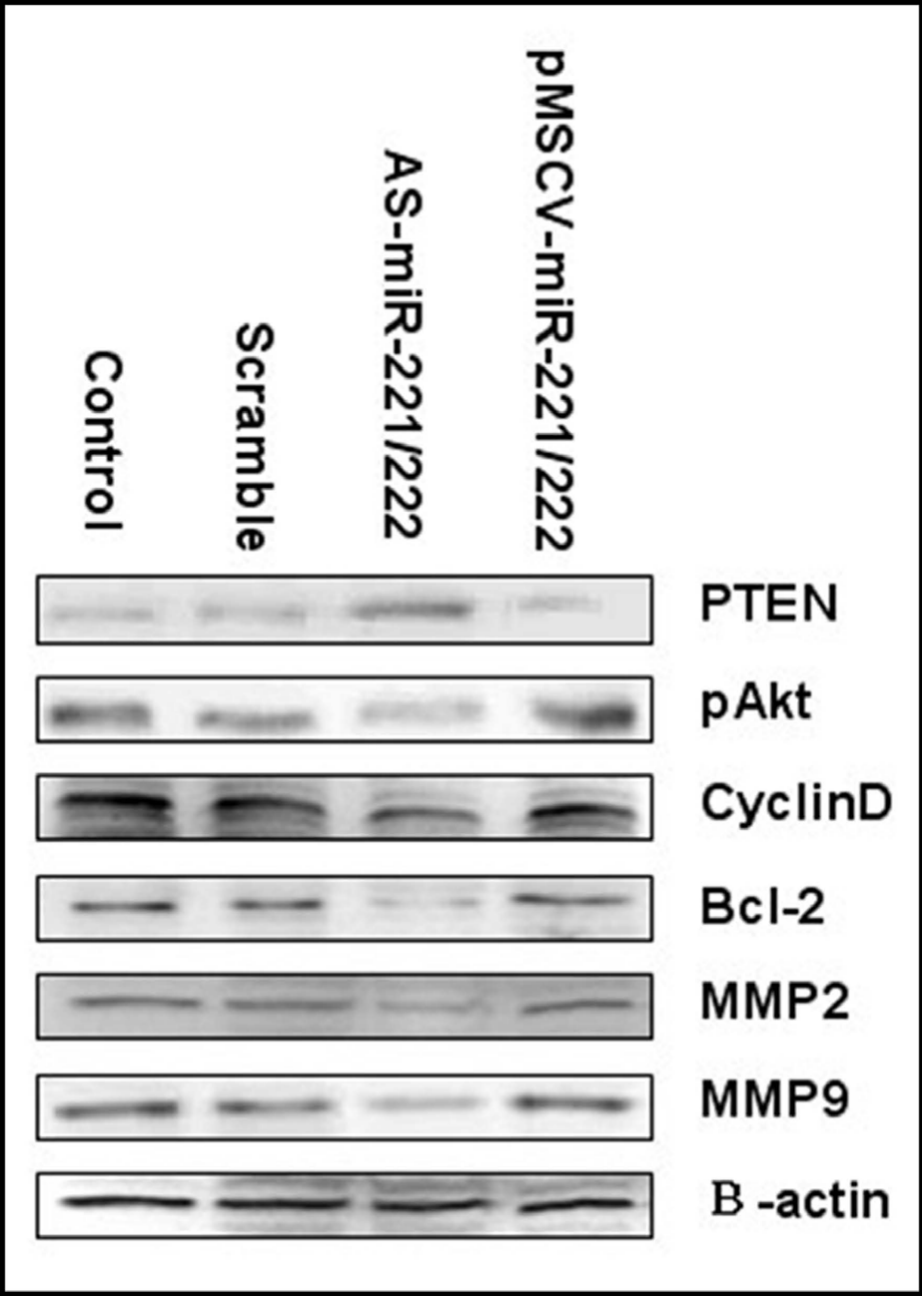
**Impact of miR-221/222 on protein expression in the Akt pathway**. Western blot analysis of SGC7901 cell lysates following genetic modulation of miR-221/222 expression with AS-miR-221/222 or pMSCV-miR-221/222. miR-221/222 inversely correlates with PTEN expression and positively correlates with pAkt, cyclinD, Bcl-2, and MMP2/9. β-actin was used as negative control.

PTEN is a tumor-suppressor gene and its role in tumor biology is well-characterized [39]. Inactivation of PTEN activates the serine/threonine protein kinase, Akt. Moreover, pAkt is a crucial protein involved in the regulation of cell-cycle progression, cell survival, apoptosis, invasion and radiosensitivity. Using Western blot analysis, we observed that the expression of PTEN was increased and the expression of pAkt was decreased in AS-miR-221/222 transfected SGC7901 cells compared to controls. Furthermore, infection of SGC7901 cells with pMSCV-miR-221/222 resulted in decreased PTEN and increased pAkt expressions (Figure 5). In addition, the expression of cyclin D, Bcl-2, MMP2 and MMP9, all of which are regulated by pAkt, were downregulated in the AS-miR-221/222 group and slightly upregulated in the pMSCV-miR-221/222 group. These data suggest that miR-221 and miR-222 impact the phenotype of SGC7901 cell by modulating the expression of PTEN and Akt phosphorylation.

## Discussion

In this study, we demonstrated that miR-221 and miR-222 regulate gastric cancer cell viability, apoptosis, cell cycle progression and invasive ability. Our data suggests that downregulation of PTEN expression and enhanced Akt phosphorylation (p-Akt) are important mediators of these cellular processes. As pAkt impacts cell proliferation, cell transit from the G0/G1 to the S phase, apoptosis, cell invasive ability, and cell radiosensitivity, downregulation of miR-221 and miR-222 expression have important biologic effects on the malignant phenotype of SGC7901 cells. These results identify AS-miR-221/222 as a potential therapeutic approach for gastric cancer via upregulation of PTEN.

PTEN functions as a tumor suppressor gene, specifically by negatively regulating the Akt/PKB signaling pathway. Genetic inactivation of PTEN is a hallmark of many cancers, including glioblastoma, endometrial and prostate cancers, and reduced expression occurs in many other tumor types. Deficiency of PTEN in the intestine has been reported to induce precancerous polyps, via the induction of formation and fission of crypts, structures located at the base of the intestine containing a rapidly dividing pool of intestinal stem cells [40]. Guo JM et al studied the microRNAs expression in primary gastric cancer tissues via microRNA microarray assay and were the first to demonstrate that PTEN was the target of miR-21 [41]; however, little is known regarding the impact of miR-221 and miR-222 on PTEN expression in gastric cancer.

miR-221 and miR-222 expression is abnormally increased in gastric cancer [42], however the mechanism by which miR-221 and miR-222 modulates tumor progression within the gut remains unknown. Here, we observed miR-221 and miR-222 upregulation in the human gastric cancer cell line SGC7901 compared with HEK293 epithelial cells, corroborating the findings of Young-kook et al [23]. miR-221 and miR-222 modulate a variety of biological functions in the SGC7901 cell, including cell proliferation, apoptosis, invasion, and radioresistance. We identified binding sites for miR-221 and miR-222 in the PTEN 3'-UTR by bioinformatics analysis, suggesting that increased expression of the miR-221/222 cluster might impact on PTEN expression. Indeed, we demonstrated that PTEN is a target gene of miR-221 and miR-222 by luciferase reporter assay. As PTEN can antagonize PI3K activity by dephosphorylating PIP3 and thereby negatively regulates the activity of Akt pathway [24,25]. Several studies suggest that the loss of the PTEN function might be the underlying factor in Akt pathway activation [43-45]; thus, our findings are consistent with an emerging body of literature.

Akt represents a subfamily of the serine/threonine kinase family [46]. It modulates the function of numerous substrates related to the cell proliferation, apoptosis and invasion and is putatively involved in the development of some cancers, such as in colon [47], prostate [48], lung [49] and thyroid cancer [50]. It has been shown that Akt activation in cancer cells can increase their invasive ability and resistance to radiotherapy [51-53]. In our study, we found that knockdown of miR-221 and miR-222 in SGC7901 cells resulted in downregulation of pAkt expression, affecting the expression of several Akt-regulated proteins including cyclin D1, Bcl-2, and MMP2/9. The malignant phenotype of the SGC7901 cells was reversed by knockdown miR-221 and miR-222, and cells were sensitized to radiation, corroborating the results of Garofalo et al [54]. As PTEN is a target of miR-221 and miR-222, and has been described previously as an important regulator of radiation sensitivity [24,55], these results suggest that increasing PTEN expression by silencing miR-221/222 could enhance the radiosensitivity of SGC7901 cells. Whether PTEN/Akt signaling is the sole target for miRNA-221/222 regulation of radiosensitivity remains unknown.

**Figure 6:**
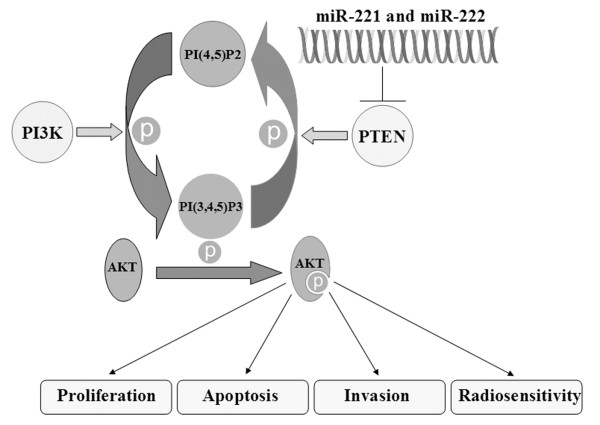
**miR-221/222-mediated regulation of signal transduction via PTEN**. Hypothetical representation of the role of miR-221 and miR-222 in regulation of Akt phosphorylation and downstream signaling of proliferation, apoptosis, invasion, and radiosensitivity via PTEN. Our data supports an essential role for miR-221/222 in the inhibition of PTEN, thereby promoting Akt phosphorylation via activated PIP3.

While our work identifies a role for PTEN in miRNA-221/222-induced biology, it remains possible other factors might be at least partially involved. Negative regulation of p27 and p57 by miRNA221/222 might also contribute to radioresistance [56], however they are more likely to contribute to cell proliferation and viability [57,58]. Moreover, TIMP3, as a target of miR-221 and miR-222, might also affect cell invasion[54]. In sum, our results suggest that inhibition of the miR221/222 cluster represents a molecular therapeutic approach that impacts on multiple genes involved in anti-tumor growth and radiosensitization, as summarized in Figure 6.

## Conclusions

The PTEN gene is an important functional target of the miR-221/222 cluster in gastric cancer cells. Modulation of miR-221/222 expression by antisense or overexpression strategies directly affected PTEN expression. At present, anti-miRNA oligonucleotides have been shown to specifically inactivate endogenous target miRNAs, although rather inefficiently [59,60]. We provide evidence that co-suppression of both miR-221 and miR-222 affects gastric cancer cell biology in vitro, and might represent a novel therapeutic strategy for gastric cancer through upregulation of PTEN expression.

## Competing interests

The authors declare that they have no competing interests.

## Authors' contributions

KCS conceived the project. ZCZ and HL carried out all the studies, analyzed the data, and wrote the first draft of the paper. ZCZ, HL, ZQY, ZAL, FYC, YX, WGX, JZF, PPY and KCS were involved in experimental design. ZQY provided guidance with the study and assisted with the manuscript draft. PPY and KCS helped in carrying out the experiments. ZAL, FYC and YX carried out the Northern blot and Western blot analysis. FYC carried out the cell viability and apoptosis assay. YX carried out the cell cycle and invasion assay. WGX carried out the clonogenic and luciferase reporter assay. JZF conducted analyses of expression data. All authors read and approved the final manuscript.

## Pre-publication history

The pre-publication history for this paper can be accessed here:

http://www.biomedcentral.com/1471-2407/10/367/prepub
